# Long non‐coding RNA SOX21‐AS1 enhances the stemness of breast cancer cells via the Hippo pathway

**DOI:** 10.1002/2211-5463.13015

**Published:** 2020-12-01

**Authors:** Lanzhen Li, Dongmei Meng, Ruiqing Wang

**Affiliations:** ^1^ Department of General Surgery Linyi People's Hospital Lanshan District, Linyi China; ^2^ College of Pharmacy Heze University Heze China; ^3^ Department of Breast Surgery Linyi People's Hospital Lanshan District, Linyi China

**Keywords:** breast cancer stem cell, Hippo pathway, lncRNA SOX21‐AS1, migration, proliferation, stemness

## Abstract

Breast cancer stem cells (BCSCs) have high tumorigenicity and invasiveness, which contributes to recurrence and metastasis. The long non‐coding RNA SOX21‐AS1 has been previously reported to modulate the properties of breast cancer stem cells via targeting SOX2, although the underlying molecular mechanisms remain unclear. To investigate this issue, we first confirmed that the expression level of SOX21‐AS1 is increased in breast cancer tissues and cell lines (MCF‐7, MDA‐MB‐231, CSC‐MCF‐7, CSC‐MDA‐MB‐231), especially in BCSCs. We demonstrated that SOX21‐AS1 promotes the stemness of CSC‐MCF‐7 cells through western blot detection of stemness‐related proteins, as well as side population and sphere formation assays. Overexpression of SOX21‐AS1 enhanced the proliferation, migration and invasion of CSC‐MCF‐7 cells. We also observed that SOX21‐AS1 inhibited the Hippo pathway. SOX21‐AS1 enhanced the stemness, migration and invasion of CSC‐MCF‐7 cells by increasing the nuclear localization of YAP and decreasing the level of pYAP. Overall, we conclude that SOX21‐AS1 may promote the stemness viability, proliferation, migration and invasion of BCSCs by inhibiting the Hippo pathway. Our findings provide insights into potential biomarkers and prognostic measures for the treatment of breast cancer.

## Graphical abstract

Previous reports have confirmed that long non‐coding RNA SOX21‐AS1 could regulate the properties of breast cancer stem cells (BCSCs), although the underlying molecular mechanism is unclear. We verified that SOX21‐AS1 was highly expressed in breast cancer tissues and cell lines, especially in BCSCs. SOX21‐AS1 could promote the stemness viability, proliferation, migration and invasion of BCSCs by inhibiting the Hippo pathway.

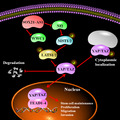

**Abbreviations**
BCSC: breast cancer stem cell
CCK‐8: cell counting kit‐8
DMEM: Dulbecco’s modified Eagle’s medium
pcDNA3.1‐SOX21‐AS1: SOX21‐AS1 overexpression vector
qRT‐PCR: quantitative reverse transcriptase PCR
si‐SOX21‐AS1#1: siRNA1 against SOX21‐AS1
si‐SOX21‐AS1#2: siRNA2 against SOX21‐AS1
SP: side population

## Introduction

Breast cancer is a type of malignancy and the global leading cause of cancer‐related death in women [1]. Studies have confirmed that stem cell‐like cell populations exist in tumor tissues [2, 3, 4]. Breast cancer stem cells (BCSCs) with self‐renewal ability and multidirectional differentiation potential in breast cancer tissues are closely associated with the occurrence, development, post‐treatment metastasis and the relapse of breast cancer [5, 6].

Long non‐coding RNA (lncRNA) is a class of RNA transcript that consists of more than 200 nucleotides and does not encode proteins [7]. Increasing evidence shows that lncRNA plays an important role in cell differentiation, proliferation and apoptosis [8]. At the same time, the role of lncRNA dysregulation in tumorigenesis and development has attracted more and more attention. Recent studies have shown that lncRNA SOX21‐AS1 is located at chromosome 13q32.1 and transcribed into a 2986 nucleotide transcript, which exerts important functions in tumorigenesis and maintenance of tumor stem cell characteristics [9]. For example, SOX21‐AS1 modulates breast cancer stem cells properties and carcinogenesis via targeting SOX2 [10]. Although there is evidence that SOX21‐AS1 is involved in the proliferation, invasion and maintenance of the stemness of BCSCs [10, 11, 12], the underlying molecular mechanisms remain unclear.

Research has shown that the Hippo signaling pathway has important functions in organ size regulation, tissue regeneration, cancer development and stem cell function as a result of the regulation of cell proliferation, apoptosis and stem cell self‐renewal ability [13, 14]. In detail, the Hippo signaling pathway is a kinase cascade. Upon the induction of the Hippo signaling pathway by the activators WWC1 and Nf2 [15], the MST1/2 kinase forms a complex with SAV1 and phosphorylates LAST1/2, and the phosphorylated LAST1/2 kinase then phosphorylates the key downstream protein effectors of the Hippo signaling pathway, YAP and TAZ. pYAP can bind to proteins in the cytoplasm and stay in the cytoplasm, causing ubiquitination and degradation, thereby inhibiting the functions of YAP with respect to promoting growth, resisting apoptosis and maintaining stemness [16]. By contrast, when the Hippo signaling pathway is inhibited, YAP can be transported to the nucleus to bind to transcription factors such as TEAD1‐4, thereby inducing gene expression, which promotes cell proliferation and maintains cell stemness [17]. The Hippo pathway maintains the characteristics of cancer stem cells and SOX21‐AS1 can promote the development of cancer stem cells, although whether SOX21‐AS1 acts on BCSC through this pathway is unknown. Accordingly, the present study aimed to investigate the potential mechanism of SOX21‐AS1 on BCSCs and to provide potential therapeutic targets for the treatment of breast cancer.

## Materials and methods

### Tissue sample collection

Breast cancer tissues and the tumor‐adjacent non‐tumor tissues were collected from Linyi People’s Hospital in Shandong Province. Tumor‐adjacent, non‐tumorous tissues were collected ≥ 3 cm away from the cancer margin. The patients did not receive any chemoradiotherapy before surgical resection. This study was approved by the Ethics Committee of Linyi People's Hospital. The clinical features of the patients are listed in Table 1. Fresh tissues were sent for western blot and quantitative reverse transcriptase PCR (qRT‐PCR) analysis immediately after resection or immediately frozen using liquid nitrogen. The study was granted permission by the Ethics Committee of Linyi People’s Hospital and all patients or their guardians provided their written informed consent for inclusion in the study. The study methodologies conformed to the standards set by the Declaration of Helsinki.

**Table 1:** Clinicopathological characteristics associated with SOX21‐AS1 expression in 52 breast cancer patients.

Parameters	Number	lncRNA SOX21‐AS1		*P*‐value
		Low	High	
*Age (years)*				
< 48	25	12	13	NS
≥ 48	27	11	16	
*Tumor size*				
< 2.5 cm	33	17	15	NS
≥ 2.5 cm	19	9	10	
*Estrogen receptor status*				
Positive	23	11	12	NS
Negative	29	12	17	
*Distant metastases*				
Yes	24	7	17	0.025^*^
No	28	15	13	
*Lymphatic metastases*				
Positive	27	10	17	0.019^*^
Negative	25	11	14	
*TNM stage*				
I–II	22	9	11	0.023^*^
III–IV	30	11	19	

*P < 0.05 indicates a statistical difference.

### Cell culture

Normal human breast epithelial cell line (MCF‐10A) and human breast cancer cell lines (MCF‐7, MDA‐MB‐231) were purchased from the American Type Culture Collection (Manassas, VA, USA). All three cell lines were successfully cultivated in Dulbecco’s modified Eagle’s medium (DMEM) containing 10% fetal bovine serum at 37 °C with 5% CO_2_. The BCSCs CSC‐MCF‐7 and CSC‐MDA‐MB‐231 were cultured and selected as described previously [18, 19].

### Stem cell isolation

The cells were cultured in serum‐free DMEM medium containing 2% B27, 20 ng·mL^−1^ epidermal growth factor, 5 mg·L^−1^ insulin and 4 g·L^−1^ BSA in an ultra‐low adherent plate and cultured at 37 °C in a 5% CO_2_ humidified incubator for 15–20 days.

### Cell transfection

Oligonucleotides sequences were synthesized and provided by Gene Pharma (Shanghai, China), including siRNA1 against SOX21‐AS1 (si‐SOX21‐AS1#1), siRNA2 against SOX21‐AS1 (si‐SOX21‐AS1#2) and si‐NC. The construction of the SOX21‐AS1 gene into pcDNA3.1 vector was performed by Shanghai Han Biotechnology Co., Ltd (Shanghai, China) to overexpress SOX21‐AS1 (pcDNA3.1‐SOX21‐AS1) and the empty pcDNA3.1 vector served as the control group. When cell confluency reached 70–80%, pcDNA3.1‐SOX21‐AS1, pcDNA3.1, si‐SOX21‐AS1#1 or si‐SOX21‐AS1#2 were transfected into CSC‐MCF‐7 cells using Lipofectamine 2000 (Invitrogen, Carlsbad, CA, USA) in accordance with the manufacturer's instructions. Forty‐eight hours after transfection, the cells were collected for subsequent analysis. The siRNA sequences were: 5'‐AACAGAAACAGAGGCUUCUCGCAUU‐3' (sense) and 5'‐AAUGCGAGAAGCCUCUGUUUCUGUU‐3' (antisense) for siRNA#1 and 5'‐CAGUUAACUUACAGUGUCUCACUUA3‐' (sense) and 5'‐UAAGUGAGACACUGUAAGUUAACUG‐3' (antisense) for siRNA#2.

### Western blot analysis

Total proteins were extracted from cells using RIPA lysis buffer (Beyotime, Shanghai, China). Each sample was separated by 10% SDS/PAGE and transferred to a poly(vinylidene difluoride) membrane (Millipore, Darmstadt, Germany). The membrane was blocked with fat‐free milk for 1 h and incubated with primary antibodies overnight at 4 °C, including SOX2 (dilution 1: 1000), OCT4 (dilution 1: 500), ABCG2 (dilution 1: 1000), NANOG (dilution 1: 1000), ALDHA1 (dilution 1: 200), WWC1 (dilution 1: 500), Nf2 (dilution 1: 500), MST1/2 (dilution 1: 500), pMST1/2 (pMST1/2), pLATS1/2 (dilution 1: 500), LATS1/2 (dilution 1: 500), YAP (dilution 1: 500), pYAP (dilution 1: 500) and GAPDH (dilution 1: 2000), which were all purchased from Thermo Fisher (Waltham, MA, USA). Next, the primary antibodies were washed using Tris‐buffered saline. The corresponding secondary antibodies were added and incubated at 37 °C for 1 h. All secondary antibodies were purchased from Thermo Fisher. Finally, the results were detected using an ECL DETECTION KIT (Beyotime).

### qRT‐PCR

Total RNA was extracted from tissues or cultured cells through the application of TRIzol reagent (Invitrogen). Reverse transcription reactions were performed with a Takara RNA PCR Kit (Takara, Shiga, Japan). qRT‐PCR was performed using a SYBR Green detection system (Takara). The primer sequences were: SOX21‐AS1 forward: 5'‐CGTGAAAGTGCCCCAATAGGTAGGTTCACA‐3', SOX21‐AS1 reverse: 5'‐TGGGGAATCTGTCTGTGTATCATTGGTT‐3', GAPDH forward: 5'‐TCGACAGTCAGCCGCATCTTCTTT‐3', GAPDH reverse: 5'‐ACCAAATCCGTTGACTCCGACCTT‐3'. The housekeeping gene GAPDH was used as an internal reference. The 2^−ΔΔCt^ method was applied to calculate the relative expression of the transcripts.

### Side population (SP) assay

For the SP assay, cells were suspended at a density of 1 × 10^6^ cells·mL^−1^ and then incubated with 5 μg·mL^−1^ Hoechst 33342 (Sigma‐Aldrich, St Louis, MO, USA) at 37 °C for 60 min. Then, 10 μm Verapamil (Sigma‐Aldrich) was used as the blocker. Then, the cells were analyzed using a flow cytometer (Thermo Fisher).

### Sphere formation assay

For sphere‐forming assay, cells were seeded into the 48‐well ultra‐low attachment plate (Corning Inc., Corning, NY, USA) in serum‐free DMEM or RPMI 1640. After 7 days of culture in the medium supplemented with B27 (dilution 1:50; Invitrogen), 20 ng·mL^−1^ epidermal growth factor (Invitrogen) and 20 ng·mL^−1^ basic fibroblast growth factor (Invitrogen), the number of spherical cells were observed under a stereomicroscope (Olympus, Tokyo, Japan).

### Clone formation assay

Briefly, CSC‐MCF‐7 cells were trypsinized into single‐cell suspensions and seeded into six‐well plates (500 cells per well) and then cultured in DMEM containing 10% fetal bovine serum for 14 days. The medium was replaced every 3 days. On day 14, the cells were fixed with 4% paraformaldehyde for 15 min and then stained using 1% crystal violet. The specific number of colonies was calculated manually.

### Cell counting kit‐8 (CCK‐8) assay

Cell viability was determined using a CCK‐8 kit (Dojindo Laboratories, Kumamoto, Japan). Briefly, CSC‐MCF‐7 cells were seeded into a 96‐well plate and incubated for different times (0, 12, 24, 48 and 72 h). Next, 10 μL of CCK‐8 solution was added into each well and incubated at 37 °C for 4 h. Subsequently, absorbance was detected at 450 nm using SpectraMax 250 spectrophotometer (Molecular Devices, Sunnyvale, CA, USA).

### Wound healing assay

After the transfected cells formed a monolayer, a 200‐μL pipette tip was used to scratch a wound and the detached cells were washed away. The cells were cultured at 37 °C for 48 h. Scratch wound healing was observed and monitored by microscopy (XSP‐11CC; Caikon, Shanghai, China). All wound area measurements were evaluated using imagej (NIH, Bethesda, MD, USA). The cell migration area was calculated as: (*S*_0 _– *S*_48_)/*S*_0_ × 100%, where *S*_0_ was the initial wound area and *S*_48_ was the wound area after 48 h.

### Transwell assay

The migration and invasion of cells were examined using the transwell assay. For migration assay, CSC‐MCF‐7 cells (5 × 10^4^ per well) were suspended in 200 mL of DMEM without serum and seeded into the chamber. For invasion assays, the Transwell membrane was coated with Matrigel (BD Biosciences, Frankline Lakes, NJ, USA). Twenty‐four hours after incubation, the non‐invaded cells were removed from the upper surface of the membrane and the cells that penetrated the pore were fixed and then stained with crystal violet.

### Flow cytometry analysis

Briefly, 5 × 10^5^ cells were resuspended with NaCl/Pi containing 2% fetal bovine serum. Then, the cells were incubated in dark for 20 min with fluorescence‐labeled antibodies IgG1‐pe/IgG‐FITC, CD44‐FITC/IgG1‐PE, CD24‐PE/IgG2‐FITC and CD44‐FITC/CD24‐PE. Finally, the cells were resuspended in 500 mL of fluorescence‐activated cell sorting buffer and analyzed using flow cytometer (Thermo Fisher). CD44^+^/CD24^−^ is the most commonly used marker for sorting BCSC. The higher the ratio of CD44^+^/CD24^−^, the stronger the stemness characteristics of the cells.

### Statistical analysis

All data are reported as the mean ± SD). spss, version 20.0 software (IBM Corp., Armonk, NY, USA) was used for statistical analysis. Differences between groups were measured by Student′s *t*‐test or one‐way analysis of variance. *P* < 0.05 was considered statistically significant. All experiments were performed at least three times.

## Results

### SOX21‐AS1 is highly expressed in breast cancer tissues and cell lines

**Fig. 1:**
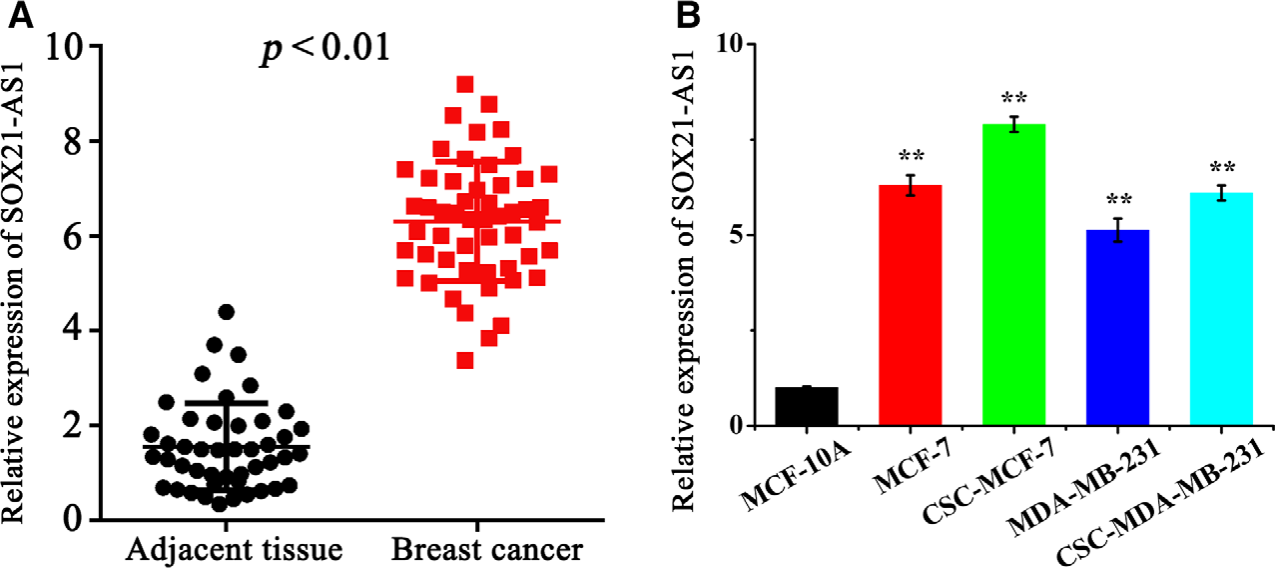
(A) qRT‐PCR was performed to examine SOX21‐AS1 expression in 52 pairs of breast cancer tissues and paired adjacent non‐tumor tissue. (B) qRT‐qPCR was performed to examine SOX21‐AS1 expression in normal breast cancer cells (MCF‐10A) and different breast cancer cell lines (MCF‐7, MDA‐MB‐231, CSC‐MCF‐7 and CSC‐MDA‐MB‐231). The results are reported as the mean ± SD of three experiments. *P < 0.05; **P < 0.01 (Student′s*t*‐test).

The expression level of SOX21‐AS1 in clinical breast cancer samples and cultured cell lines was detected by qRT‐PCR. The results are shown in Fig. 1. Compared to the tumor‐adjacent normal samples and human breast epithelial cell line, SOX21‐AS1 was highly expressed in clinical breast cancer samples and cultivated cell lines. Moreover, the expression level of SOX21‐AS1 in CSC‐MCF‐7 and CSC‐MDA‐MB‐231 cells was higher than that of MCF‐7 and MDA‐MB‐231 cells, among which the expression level of SOX21‐AS1 in CSC‐MCF‐7 was the highest. Therefore, CSC‐MCF‐7 cells were selected for the subsequent experiments.

### SOX21‐AS1 promotes the tumor stem cell properties of CSC‐MCF‐7

**Fig. 2:**
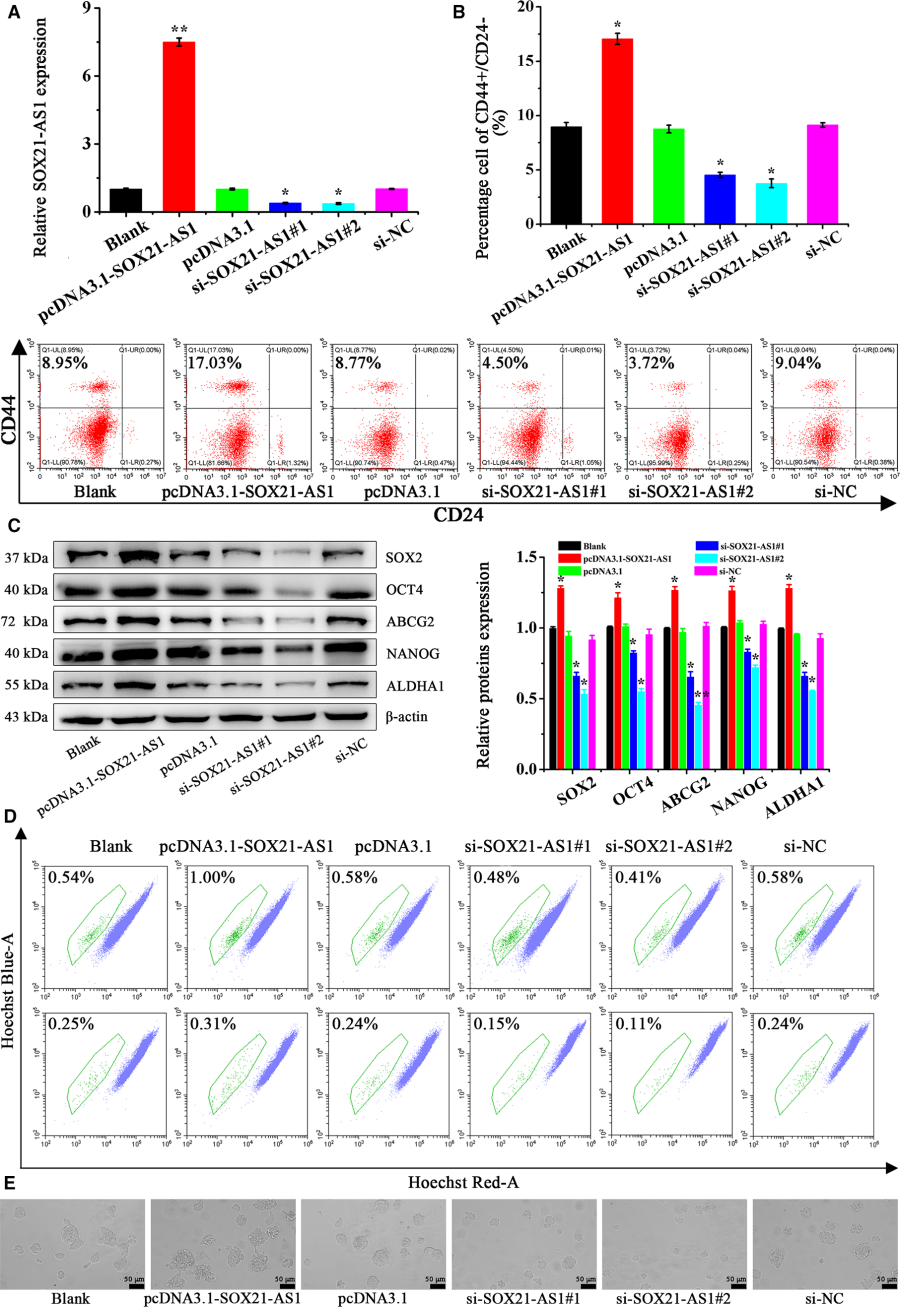
(A) qRT‐PCR was used to analyze the expression of SOX21‐AS1 in CSC‐MCF‐7 cells after different treatments (Blank, pcDNA3.1‐SOX21‐AS1, pcDNA3.1, si‐SOX21‐AS1#1, si‐SOX21‐AS1#2, si‐NC). (B) The ratio of CD44^+^/CD24^–^in CSC‐MCF‐7 cells after different treatments (Blank, pcDNA3.1‐SOX21‐AS1, pcDNA3.1, si‐SOX21‐AS1#1, si‐SOX21‐AS1#2, si‐NC) was detected by flow cytometer analysis. (C) The expression of CSC‐MCF‐7 cell stemness‐related proteins after different treatments (Blank, pcDNA3.1‐SOX21‐AS1, pcDNA3.1, si‐SOX21‐AS1#1, si‐SOX21‐AS1#2, si‐NC) was detected by western blot analysis. (D) The proportion of SP in CSC‐MCF‐7 cells after different treatments (Blank, pcDNA3.1‐SOX21‐AS1, pcDNA3.1, si‐SOX21‐AS1#1, si‐SOX21‐AS1#2, si‐NC) was detected by flow cytometer analysis. (E) Sphere formation ability in CSC‐MCF‐7 cells after different treatments (Blank, pcDNA3.1‐SOX21‐AS1, pcDNA3.1, si‐SOX21‐AS1#1, si‐SOX21‐AS1#2, si‐NC) was detected using a stereomicroscope. Scale bars = 50 μm (*n* = 3). The results are reported as the mean ± SD of three experiments. *P < 0.05; **P < 0.01 (Student′s*t*‐test).

To further examine the effect of SOX21‐AS1 on CSC‐MCF‐7 cells, we performed additional analyses. The CSC‐MCF‐7 cells were divided into different groups (Blank, pcDNA3.1‐SOX21‐AS1, pcDNA3.1, si‐SOX21‐AS1#1, si‐SOX21‐AS1#2, si‐NC). Transfection efficiency was detected via qRT‐PCR. The transfection efficiencies of each group are shown in Fig. 2A.

Next, the effect of SOX21‐AS1 on the stemness of CSC‐MCF‐7 cells was determined by flow cytometry analysis. In Fig. 2B, the ratio of CD44^+^/CD24^–^ in the Blank, pcDNA3.1‐SOX21‐AS1, pcDNA3.1, si‐SOX21‐AS1#1, si‐SOX21‐AS1#2 and si‐NC groups was 8.96 ± 0.40%, 16.97 ± 0.51%, 8.72 ± 0.35%, 4.53 ± 0.26%, 3.79 ± 0.43%, 9.06 ± 0.21%, respectively. The results of flow cytometry showed that the ratio of CD44^+^/CD24^–^ in the pcDNA3.1‐SOX21‐AS1 group was the highest, followed by the Blank, pcDNA3.1 and si‐NC groups, and there was no significant difference with respect to the ratio of CD44^+^/CD24^–^ in these three groups. The ratio of CD44^+^/CD24^–^ was lower in the si‐SOX21‐AS1#1 group and the si‐SOX21‐AS1#2 group had the lowest ratio. From the results of flow cytometry, we inferred that SOX21‐AS1 overexpression could promote the stemness of BCSC. Western blot analysis was used to detect the expression levels of stemness relative proteins, and the results are shown in Fig. 2C. The expression levels of SOX2, OCT4, ABCG2, NANOG and ALDHA1 proteins in the pcDNA3.1‐SOX21‐AS1 group were significantly upregulated, followed by the Blank, pcDNA3.1 and si‐NC groups, whereas, in the si‐SOX21‐AS1#1 and si‐SOX21‐AS1#2 groups, the expression of SOX2, OCT4, ABCG2, NANOG and ALDHA1 was significantly inhibited. More importantly, the results of SP and sphere formation assays are shown in Fig. 2D,E. We found that, compared to the Blank, pcDNA3.1 and si‐NC groups, overexpression of SOX21‐AS1 in the pcDNA3.1‐SOX21‐AS1 group increased the percentage of SP and the sphere formation ability of the cells. However, after silencing the expression of SOX21‐AS1, the percentage of SP and the cell sphere formation ability were decreased in the si‐SOX21‐AS1#1 and si‐SOX21‐AS1#2 groups.

**Fig. 3:**
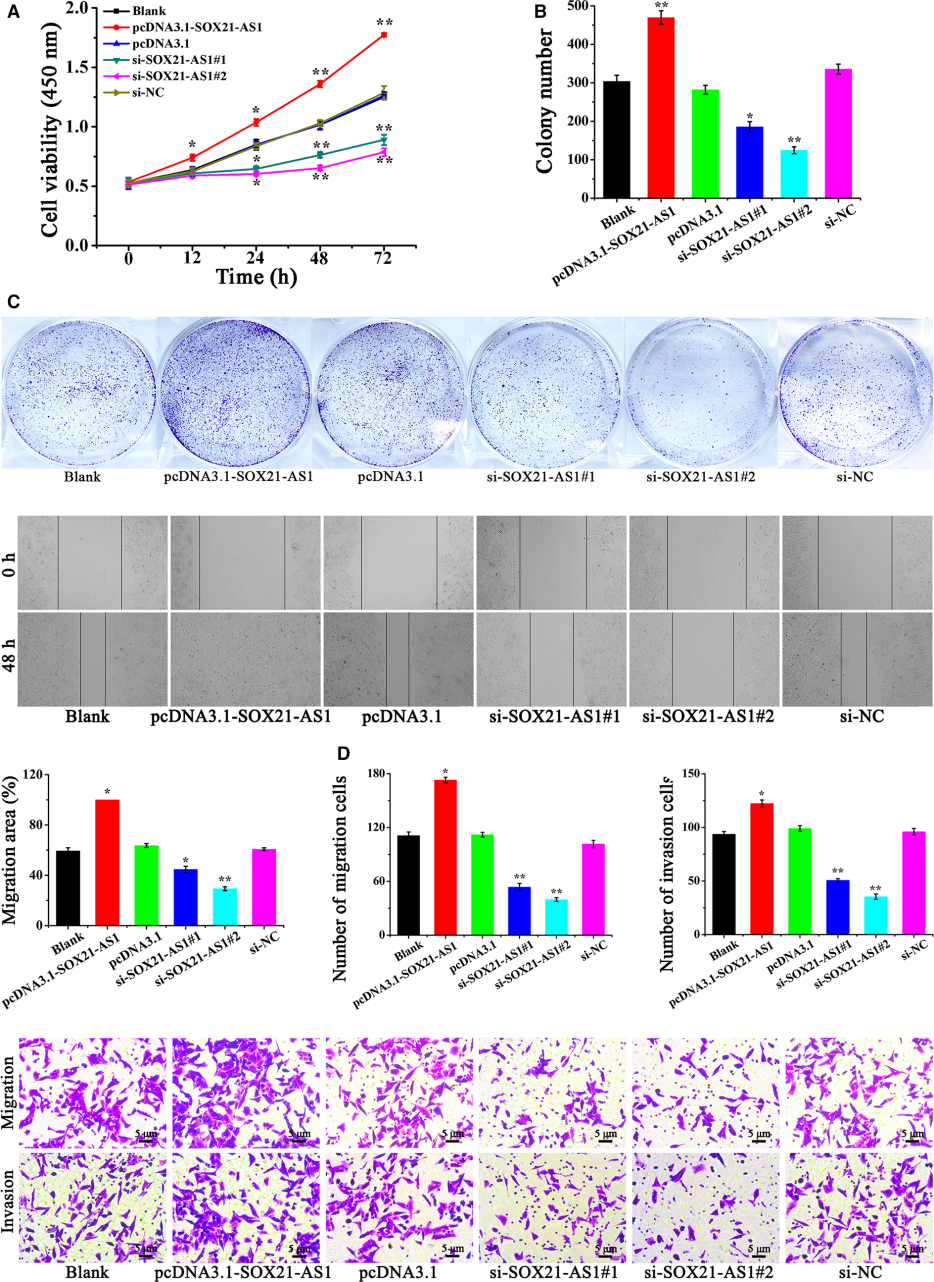
(A) The cell viability of CSC‐MCF‐7 cells after different treatments (Blank, pcDNA3.1‐SOX21‐AS1, pcDNA3.1, si‐SOX21‐AS1#1, si‐SOX21‐AS1#2, si‐NC) was detected by a CCK‐8 assay. (B) The proliferation ability of CSC‐MCF‐7 cells after different treatments (Blank, pcDNA3.1‐SOX21‐AS1, pcDNA3.1, si‐SOX21‐AS1#1, si‐SOX21‐AS1#2, si‐NC) was detected by a clone formation assay. (C) The migration ability of CSC‐MCF‐7 cells after different treatments (Blank, pcDNA3.1‐SOX21‐AS1, pcDNA3.1, si‐SOX21‐AS1#1, si‐SOX21‐AS1#2, si‐NC) was detected by a wound healing assay. (D) A transwell assay was applied to detect the migration and invasion of CSC‐MCF‐7 cells after different treatments (Blank, pcDNA3.1‐SOX21‐AS1, pcDNA3.1, si‐SOX21‐AS1#1, si‐SOX21‐AS1#2, si‐NC). Scale bars = 5 μm (*n* = 3). The results are reported as the mean ± SD of three experiments. *P < 0.05; **P < 0.01 (Student′s*t*‐test).

Cell stemness is closely associated with the proliferation, migration and invasion of tumor cells. Therefore, we conducted experiments to determine the influence of SOX21‐AS1 on CSC‐MCF‐7 cells. First, we explored the effect of SOX21‐AS1 on the cell viability of CSC‐MCF‐7 cells using the CCK‐8 assay. As shown in Fig. 3A, overexpression of SOX21‐AS1 in the pcDNA3.1‐SOX21‐AS1 group significantly enhanced the cell viability of CSC‐MCF‐7 cells compared to the Blank, pcDNA3.1 and si‐NC groups, whereas cell viability in the si‐SOX21‐AS1#1 and si‐SOX21‐AS1#2 groups was significantly decreased. The results of the CCK‐8 assay showed that SOX21‐AS1 could increase the viability of CSC‐MCF‐7 cells. Subsequently, the effect of SOX21‐AS1 on the proliferation of CSC‐MCF‐7 cells was detected by a clone formation assay. The results shown in Fig. 3B indicate that the number of colonies in the pcDNA3.1‐SOX21‐AS1 group was significantly increased compared to the Blank, pcDNA3.1 and si‐NC groups, whereas those in the si‐SOX21‐AS1#1 and si‐SOX21‐AS1#2 groups were significantly decreased. Among them, the number of colonies in si‐SOX21‐AS1#2 group was the lowest. From these results, we found that SOX21‐AS1 promoted the proliferation of CSC‐MCF‐7 cells.

To investigate the effect of SOX21‐AS1 on the migration ability of CSC‐MCF‐7 cells, a wound healing assay was performed. The outcomes are shown in Fig. 3C. The overexpression of SOX21‐AS1 in pcDNA3.1‐SOX21‐AS1 group significantly accelerated the migration rate of CSC‐MCF‐7 cells, whereas the migration rate of the si‐SOX21‐AS1#1 and si‐SOX21‐AS1#2 groups was significantly reduced compared to the Blank, pcDNA3.1 and si‐NC groups. These outcomes indicated that SOX21‐AS1 could increase the migration ability of CSC‐MCF‐7.

Next, the effect of SOX21‐AS1 on the invasion and migration ability of CSC‐MCF‐7 cells was further verified through a transwell assay, and the outcomes are shown in Fig. 3D. Compared to Blank, pcDNA3.1 and si‐NC groups, the cell migration and invasion rate of the pcDNA3.1‐SOX21‐AS1 group increased significantly, whereas those of the si‐SOX21‐AS1#1 and si‐SOX21‐AS1#2 groups decreased significantly. The percentage of migrated cells in the Blank, pcDNA3.1‐SOX21‐AS1, pcDNA3.1, si‐SOX21‐AS1#1, si‐SOX21‐AS1#2 and si‐NC groups was 111.21 ± 4.01%, 173.16 ± 3.05%, 112.36 ± 2.65%, 53.73 ± 4.13%, 39.68 ± 2.08% and 101.56 ± 4.08%, respectively. Furthermore, the percentage of invaded cells in the Blank, pcDNA3.1‐SOX21‐AS1, pcDNA3.1, si‐SOX21‐AS1#1, si‐SOX21‐AS1#2 and si‐NC groups was 93.57 ± 2.51%, 122.35 ± 3.21%, 98.96 ± 2.64%, 50.66 ± 1.52%, 35.41 ± 2.47% and 95.94 ± 3.04%, respectively. In conclusion, the results of the wound healing and transwell assays showed that the upregulation of SOX21‐AS1 promoted the migration and invasion abilities of CSC‐MCF‐7 cells. Overall, the results outlined above revealed that SOX21‐AS1 could maintain cell stemness, as well as promote the proliferation, migration and invasion abilities of CSC‐MCF‐7 cells.

### SOX21‐AS1 promotes stemness, as well as the proliferation, migration, invasion of CSC‐MCF‐7 cells, by inhibiting the Hippo signaling pathway

**Fig. 4:**
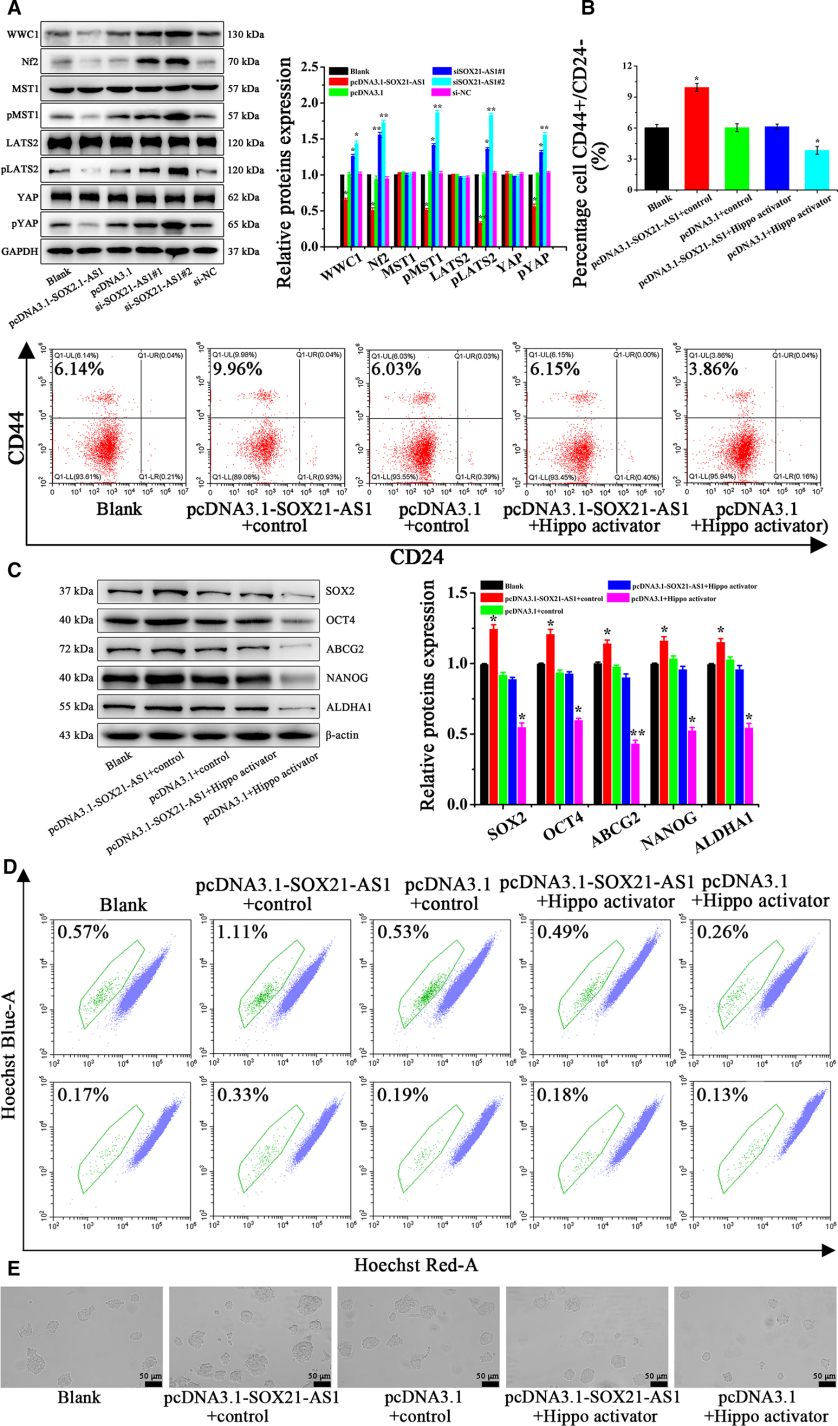
(A) The expression of the Hippo signal pathway‐related proteins in differently treated CSC‐MCF‐7 cells (Blank, pcDNA3.1‐SOX21‐AS1, pcDNA3.1, si‐SOX21‐AS1#1, si‐SOX21‐AS1#2, si‐NC) was detected by western blot analysis. (B) The ratio of CD44^+^/CD24^–^in CSC‐MCF‐7 cells after different treatments (Blank, pcDNA3.1‐SOX21‐AS1 + control, pcDNA3.1 + control, pcDNA3.1‐SOX21‐AS1 + Hippo activator, pcDNA3.1 + Hippo activator) was detected by flow cytometer analysis. (C) The expression of CSC‐MCF‐7 cell stemness‐related proteins after different treatments (Blank, pcDNA3.1‐SOX21‐AS1 + control, pcDNA3.1 + control, pcDNA3.1‐SOX21‐AS1 + Hippo activator, pcDNA3.1 + Hippo activator) was detected by western blot analysis. (D) The proportion of SP in CSC‐MCF‐7 cells after different treatments (Blank, pcDNA3.1‐SOX21‐AS1 + control, pcDNA3.1 + control, pcDNA3.1‐SOX21‐AS1 + Hippo activator, pcDNA3.1 + Hippo activator) was detected by flow cytometer analysis. (E) Sphere formation ability in CSC‐MCF‐7 cells after different treatments (Blank, pcDNA3.1‐SOX21‐AS1 + control, pcDNA3.1 + control, pcDNA3.1‐SOX21‐AS1 + Hippo activator, pcDNA3.1 + Hippo activator) was detected using a stereomicroscope. Scale bars = 50 μm (*n* = 3). The results are reported as the mean ± SD of three experiments. *P < 0.05; **P < 0.01 (Student′s*t*‐test).

At first, western blot analysis was applied to detect the changes in the expression of related proteins involved in the Hippo pathway in different groups of CSC‐MCF‐7 cells (Blank, pcDNA3.1‐SOX21‐AS1, pcDNA3.1, si‐SOX21‐AS1#1, si‐SOX21‐AS1#2, si‐NC). The results are shown in Fig. 4A. Compared to the Blank, pcDNA3.1 and si‐NC groups, the expression levels of WWC1, Nf2, pMST1 and pLATS2 in the pcDNA3.1‐SOX21‐AS1 group were all decreased, and the expression level of pYAP was also significantly diminished. However, in the si‐SOX21‐AS1#1 and si‐SOX21‐AS1#2 groups, the expression levels of WWC1, Nf2, pMST1, pLATS2 and pYAP were significantly increased. Therefore, we considered that SOX21‐AS1 could affect the stemness, proliferation, migration and invasion of CSC‐MCF‐7 cells by action on the Hippo signaling pathway.

Next, the possibility that SOX21‐AS1 could promote stemness, as well as the proliferation, migration and invasion of CSC‐MCF‐7 cells, by inhibiting the Hippo signaling pathway was investigated via rescue experiments. YAP, as a co‐transcription factor, binds to the transcription factor TEAD, which makes the effect of YAP on tumor activity largely dependent on TEAD‐mediated transcription. As an activator of the Hippo pathway, sitagliptin can significantly reduce the nuclear localization of YAP, increase the pYAP level and decrease the expression of TEAD4. Flow cytometry was conducted to verify the effect of SOX21‐AS1 on the stemness of CSC‐MCF‐7 cells through the Hippo pathway. Cells were divided into five groups (Blank, pcDNA3.1‐SOX21‐AS1 + control, pcDNA3.1 + control, pcDNA3.1‐SOX21‐AS1 + Hippo activator, pcDNA3.1 + Hippo activator) and the results are shown in Fig. 4B. The CD44^+^/CD24^–^ ratio in the Blank, pcDNA3.1‐SOX21‐AS1 + control, pcDNA3.1 + control, pcDNA3.1‐SOX21‐AS1 + Hippo activator and pcDNA3.1 + Hippo activator groups was 6.11 ± 0.31%, 9.91 ± 0.37%, 6.02 ± 0.35%, 6.11 ± 0.25% and 3.83 ± 0.38%, respectively. In the pcDNA3.1 + Hippo activator group, the CD44^+^/CD24^–^ ratio of CSC‐MCF‐7 cells decreased significantly, followed by the Blank, pcDNA3.1 + control and pcDNA3.1‐SOX21‐AS1 + Hippo activator groups, whereas, in the pcDNA3.1‐SOX21‐AS1 + control group, the CD44^+^/CD24^–^ ratio was increased significantly. Compared to the pcDNA3.1‐SOX21‐AS1 + control group, the effect of SOX21‐AS1 on the stemness of CSC‐MCF‐7 cells in the pcDNA3.1‐SOX21‐AS1 + Hippo activator group was weakened as a result of the addition of Hippo activator. Subsequently, the stemness relative proteins were detected by western blot analyis, and the results are shown in Fig. 4C. The expression levels of SOX2, OCT4, ABCG2, NANOG and ALDHA1 were significantly upregulated in the pcDNA3.1‐SOX21‐AS1 + control group and significantly decreased in the pcDNA3.1 + Hippo activator group compared to the Blank, pcDNA3.1 + control and pcDNA3.1‐SOX21‐AS1 + Hippo activator groups. Meanwhile, the effect of SOX21‐AS1 on cell stemness relative proteins was weakened in the pcDNA3.1‐SOX21‐AS1 + Hippo activator group as a result of the addition of Hippo activator compared to the pcDNA3.1‐SOX21‐AS1 + control group. Subsequently, SP and sphere formation assays were also used to verify the effect of SOX21‐AS on the stemness of CSC‐MCF‐7 cells through the Hippo pathway. Figure 4D,E shows that, in the pcDNA3.1‐SOX21‐AS1 + control group, the percentage of SP and sphere formation ability were significantly increased, whereas, in the pcDNA3.1 + Hippo activator group, these two values were significantly decreased compared to the Blank, pcDNA3.1 + control and pcDNA3.1‐SOX21‐AS1 + Hippo activator groups. It is worth noting that, compared to the pcDNA3.1‐SOX21‐AS1 + control group, the effect of SOX21‐AS1 on cell stemness was significantly reduced in the pcDNA3.1‐SOX21‐AS1 + Hippo activator group as a result of the addition of Hippo activator. Therefore, the above results confirmed that SOX21‐AS1 could promote the stemness of CSC‐MCF‐7 cells through the Hippo pathway.

**Fig. 5:**
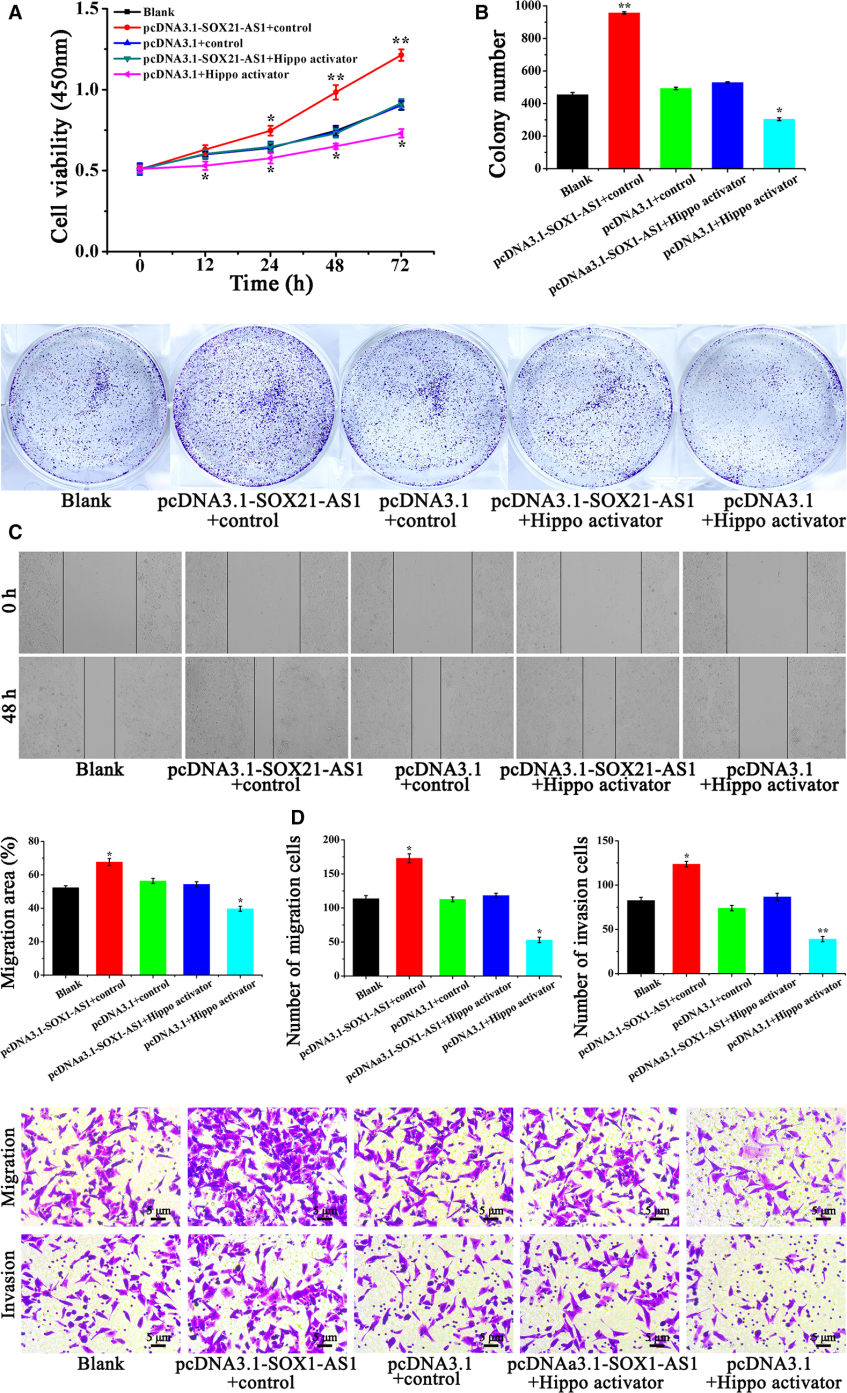
(A) Cell viability of CSC‐MCF‐7 cells after different treatments (Blank, pcDNA3.1‐SOX21‐AS1 + control, pcDNA3.1 + control, pcDNA3.1‐SOX21‐AS1 + Hippo activator, pcDNA3.1 + Hippo activator) was examined by a CCK‐8 assay. (B) The proliferation of CSC‐MCF‐7 cells after different treatments (Blank, pcDNA3.1‐SOX21‐AS1 + control, pcDNA3.1 + control, pcDNA3.1‐SOX21‐AS1 + Hippo activator, pcDNA3.1 + Hippo activator) was examined by a clone formation assay. (C) The migration ability of CSC‐MCF‐7 cells after different treatments (Blank, pcDNA3.1‐SOX21‐AS1 + control, pcDNA3.1 + control, pcDNA3.1‐SOX21‐AS1 + Hippo activator, pcDNA3.1 + Hippo activator) was detected by a wound healing assay. (D) A transwell assay was applied to detect the migration and invasion of CSC‐MCF‐7 cells after different treatments (Blank, pcDNA3.1‐SOX21‐AS1 + control, pcDNA3.1 + control, pcDNA3.1‐SOX21‐AS1 + Hippo activator, pcDNA3.1 + Hippo activator). Scale bars = 5 μm (*n* = 3). The results are reported as the mean ± SD of three experiments. *P < 0.05; **P < 0.01 (Student′s*t*‐test).

The results of the CCK‐8 assay shown in Fig. 5A demonstrated that, compared to the Blank, pcDNA3.1 + control and pcDNA3.1‐SOX21‐AS1 + Hippo activator groups, the cell viability of the pcDNA3.1 + Hippo activator group was significantly decreased, whereas that in the pcDNA3.1‐SOX21‐AS1 + control group was significantly increased. From a comparison between the pcDNA3.1‐SOX21‐AS1 + control group and the pcDNA3.1‐SOX21‐AS1 + Hippo activator group, it can be seen that the addition of Hippo activator weakened the effect of SOX21‐AS1, thus decreasing the viability of CSC‐MCF‐7 cells. These outcomes showed that SOX21‐AS1 could significantly increase the cell viability of CSC‐MCF‐7 cells through the Hippo pathway.

Figure 5B shows that the colony number of CSC‐MCF‐7 cells treated by pcDNA3.1 + Hippo activator was reduced significantly, whereas that in the pcDNA3.1‐SOX21‐AS1 + control group was significantly increased compared to the Blank, pcDNA3.1 + control and pcDNA3.1‐SOX21‐AS1 + Hippo activator groups. In the pcDNA3.1‐SOX21‐AS1 + control group, the ability of SOX21‐AS1 with respect to promoting the proliferation of CSC‐MCF‐7 cells was weakened as a result of the addition of Hippo activator compared to the pcDNA3.1‐SOX21‐AS1 + Hippo activator groups. The results of the clone formation assay indicated that SOX21‐AS1 could increase the cell proliferation of CSC‐MCF‐7 cells through the Hippo pathway.

To confirm whether SOX21‐AS1 can affect the migration of CSC‐MCF‐7 cells through the Hippo pathway, a wound healing assay was performed. The results are shown in Fig. 5C. We found that cell migration ability was the highest in the pcDNA3.1‐SOX21‐AS1 + control group, followed by the Blank, pcDNA3.1 + control and pcDNA3.1‐SOX21‐AS1 + Hippo activator groups, and, finally, the pcDNA3.1 + Hippo activator group. From a comparison between the pcDNA3.1‐SOX21‐AS1 + control group and the pcDNA3.1‐SOX21‐AS1 + Hippo activator group, we found that the addition of Hippo activator weakened the effect of SOX21‐AS1 on the promotion of CSC‐MCF‐7 migration. The above results confirmed that SOX21‐AS1 promoted the migration of CSC‐MCF‐7 cells through the Hippo pathway.

A transwell assay was applied to further confirm the effect of SOX21‐AS1 on CSC‐MCF‐7 through the Hippo pathway. Figure 5D shows that the percentage of migrated cells in the Blank, pcDNA3.1‐SOX21‐AS1 + control, pcDNA3.1 + control, pcDNA3.1‐SOX21‐AS1 + Hippo activator and pcDNA3.1 + Hippo activator groups was 113.65 ± 4.50%, 172.96 ± 6.55%, 112.57 ± 3.51%, 118.34 ± 3.21% and 52.83 ± 4.02%, respectively. Furthermore, the number of invaded cells in the Blank, pcDNA3.1‐SOX21‐AS1 + control, pcDNA3.1 + control, pcDNA3.1‐SOX21‐AS1 + Hippo activator and pcDNA3.1 + Hippo activator groups was 82.49 ± 3.51%, 123.71 ± 3.05%, 73.97 ± 3.03%, 86.52 ± 4.05% and 38.87 ± 2.96%, respectively. Compared to the the Blank, pcDNA3.1 + control and pcDNA3.1‐SOX21‐AS1 + Hippo activator groups, the highest cell migration and invasion abilities were detected in the pcDNA3.1‐SOX21‐AS1 + control group, whereas the pcDNA3.1 + Hippo activator group had the weakest ability with respect to cell migration and invasion. Compared to the pcDNA3.1‐SOX21‐AS1 + control group, the ability of SOX21‐AS1 with respect to the promotion of CSC‐MCF‐7 cell migration and invasion was reduced in the pcDNA3.1‐SOX21‐AS1 + Hippo activator group by the addition of Hippo activator. In summary, we have confirmed that SOX21‐AS1 could maintain cell stemness, as well as promote the proliferation, migration and invasion of CSC‐MCF‐7 cells, by inhibiting the Hippo pathway.

## Discussion

Breast cancer is one of the common malignant tumors in women [20]. The discovery of tumor stem cells has confirmed that they play important roles in tumor survival, proliferation, metastasis and relapse [21]. In breast cancer, lncRNAs can regulate cell proliferation, migration and invasion, as well as maintain the stemness of BCSC [22, 23, 24]. It has been documented that the lncRNA level is significantly dysregulated in breast cancer compared to normal mammary cells. Tripathi *et al*. [25] demonstrated that LINC010187 was involved in oncogenic activity by controlling AKT1 and PAWR genes. Moreover, Zhou *et al*. [26] showed that lncRNA Hh strengthened cancer stem cell generation in breast cancer via induction of the hedgehog signaling pathway. Meanwhile, the function and mechanism of lncRNA in the development and prognosis of breast cancer have been increasingly emphasized [27]. Therefore, the in‐depth exploration of lncRNA has a very important role in the early diagnosis of breast cancer, including the search for potential biomarkers and the prognosis [28]. In the present study, we investigated lncRNA SOX21‐AS1 and found that the expression of SOX21‐AS1 in breast cancer was disordered. It was confirmed that SOX21‐AS1 could promote the stemness of CSC‐MCF‐7 cells through the detection of stemness‐related proteins, as well as side population and sphere formation assays. At the same time, we also found that SOX21‐AS1 had a significant promoting effect on the proliferation, migration and invasion of CSC‐MCF‐7.

The Hippo signaling pathway inhibits cell growth [29]. In mammals, upstream membrane protein receptors of the Hippo signaling pathway receive extracellular growth‐inhibitory signals [30]. Once the extracellular growth‐inhibitory signals are sensed, they activate a series of kinase cascade phosphorylation reactions and eventually phosphorylate downstream effector YAP and TAZ [31]. Subsequently, the cytoskeleton binds to phosphorylated YAP and TAZ, which causes them to remain in the cytoplasm for degradation, thereby inhibiting their function with respect to promoting proliferation and growth, as well as inhibiting apoptosis [32]. Conversely, unphosphorylated YAP and TAZ can enter the nucleus and bind to TEADs or other transcription factors, thereby inducing the upregulated expression of genes to promote proliferation and inhibit apoptosis [33]. Accordingly, the dysregulation of the Hippo pathway plays an important role in the occurrence and development of tumors. In the present study, we confirmed that the upregulation of SOX21‐AS1 suppressed the Hippo pathway. SOX21‐AS1 promoted the stemness of CSC‐MCF‐7 cells and also enhanced the proliferation, migration and invasion of CSC‐MCF‐7 cells by increasing the nuclear localization of YAP and reducing the level of pYAP.

In summary, we found that SOX21‐AS1 could promote breast cancer stem cell stemness, proliferation, migration and invasion through the Hippo signaling pathway. The results of our study provide a reliable research basis for exploring the underlying action mechanism of SOX21‐AS1 in breast cancer and indicate a new potential direction for the clinical treatment of breast cancer.

## Conflict of interests

The authors declare that they have no conflicts of interest.

## Author contributions

RW designed the project and revised the manuscript. LL and MD acquired and analysed the data, and wrote the paper. All authors gave final approval of the version to be published, and agree to be accountable for all aspects of the work.

## Data Availability

The analyzed data sets generated during the present study are available from the corresponding author upon reasonable request.
